# Setting the Next Vital Sign Observation Interval as a Learning Objective in Simulation-Based Nursing Education: A Prospective Exploratory Observational Study

**DOI:** 10.3390/nursrep15120416

**Published:** 2025-11-26

**Authors:** Keisuke Endo, Kazumi Kubota, Kenji Karino, Rie Sato, Seiko Miura, Yasunori Ueda, Yoshiaki Iwashita

**Affiliations:** 1 Department of Nursing, Shimane University Hospital, 89-1 Enya-cho, Izumo 693-8501, Shimane, Japan; endo41@med.shimane-u.ac.jp; 2 Research Organization, Shimonoseki City University, 2-1-1 Daigaku-cho, Shimonoseki 751-8510, Yamaguchi, Japan; kubota-ka@shimonoseki-cu.ac.jp; 3 Human Resource Development Center, Hikawa Health Co-Operative Association, 4883-1 Naoe, Hikawa-cho, Izumo 699-0631, Shimane, Japan; ken2.karino@gmail.com; 4 Department of Emergency and Critical Care Medicine, Faculty of Medicine, Shimane University, 89-1 Enya-cho, Izumo 693-8501, Shimane, Japan; riesato@med.shimane-u.ac.jp; 5 Department of Radiology, Kanazawa Medical University, 1-1 Daigaku, Uchinada, Kahoku-gun 920-0293, Ishikawa, Japan; miura@kanazawa-med.ac.jp; 6 Department of Emergency Medical Science, Faculty of Health Sciences, Meiji University of Integrative Medicine, Hiyoshi-cho, Nantan 629-0392, Kyoto, Japan; y_ueda@meiji-u.ac.jp

**Keywords:** clinical deterioration, early warning score, simulation-based education, nursing clinical judgment, observation frequency

## Abstract

**Background/Objectives:** Abnormal vital signs often precede in-hospital clinical deterioration, but little is known about how nurses decide when to recheck vital signs. We examined how nurse characteristics relate to the next vital sign observation interval after detecting abnormal values and how this decision could be used as a learning objective in simulation-based education. **Methods:** In this prospective exploratory observational study at a university hospital in Japan, twenty-seven nurses used a full-body patient simulator across three scenarios: normal, low-urgency, and moderate-risk (moderately abnormal vital signs according to National Early Warning Score 2 [NEWS2] risk bands). After each assessment, participants specified in hours the interval they considered appropriate for the next vital sign observation. Nurse characteristics included years of clinical experience, advanced life support (ALS) training, and prior experiences recognizing or responding to deterioration. Mann–Whitney U tests and multiple regression were used to explore univariate and adjusted associations. **Results:** In the low-urgency scenario, ALS training was associated with shorter intervals (median 1 h vs. 3 h; *p* = 0.04). In the moderate-risk scenario, univariate analyses showed shorter intervals among nurses with greater experience and among those with ALS training (both *p* < 0.01). In adjusted models for the moderate-risk scenario, years of experience and prior experiences of recognizing and responding to deterioration were independently associated with shorter intervals (all *p* < 0.05), whereas ALS training was not. **Conclusions:** The decision to shorten observation intervals appears to reflect experiential aspects of clinical judgment. Integrating “setting the next observation interval” as an explicit learning objective in simulation may help strengthen nurses’ clinical judgment for early recognition of deterioration. As an exploratory, single-center study with a small sample and fixed scenario order, these findings should be interpreted cautiously and used to guide larger confirmatory studies and curricular design. This study was not registered.

## 1. Introduction

Early physiological abnormalities commonly precede catastrophic in-hospital events, including cardiac arrest. Classic and contemporary studies have shown that derangements such as elevated respiratory rate are frequent hours before deterioration, underscoring the actionable window for recognition and intervention [1,2,3]. Nevertheless, survival to discharge after in-hospital cardiac arrest remains limited, typically below one quarter in large cohorts and registries [4,5,6]. To bridge the gap between early signals and timely response, rapid response systems (RRS) have been implemented internationally to enable prompt expert intervention when predefined criteria are met [7]. Nurses initiate a substantial proportion of RRS activations and are pivotal in noticing change and escalating care on general wards [8,9,10].

Standardized early warning systems, notably the National Early Warning Score 2 (NEWS2), recommend observation frequencies aligned with risk strata and emphasize using clinical judgment to tailor monitoring [11]. Yet, monitoring frequency in practice often diverges from protocol as risk increases, reflecting trade-offs among workload, prioritization, and bedside judgment [12]. Digital implementations of NEWS2 can improve timeliness of observations but do not uniformly transform human decision-making around interval setting or escalation [13]. Notably, a stepped-wedge study in the UK NHS reported shorter time to the next observation after electronic NEWS2 implementation but persistent variability in interval-setting behavior [13]. Combining early warning scores with structured clinical assessment has been proposed to strengthen monitoring decisions [14], consistent with longstanding guidance that both protocol and judgment should inform physiological monitoring [15]. While early warning models demonstrate prognostic discrimination [16,17], translating risk into consistent observation behaviors remains a human factors challenge.

While our simulation isolated clinical judgment from contextual constraints, real-world monitoring intervals are continuously negotiated against workload, competing demands, and resource limitations. This human factor context likely contributes to persistent variability in interval-setting behavior observed after electronic NEWS2 implementations and underscores the need to support clinical judgment within ward work realities.

Nurses’ judgments are shaped not only by scores but also by contextual cues, frequent intentional room visits, and attention to subtle changes in condition [18]. Conceptual models of clinical judgment in nursing highlight pattern recognition, cue interpretation, and response selection as experience-dependent processes [19], and professional growth from novice to expert enables anticipatory decision-making [20]. A specific, observable decision that crystallizes these processes is the interval set for the next vital sign check after detecting abnormal values. Despite its centrality to patient safety, interval setting has received little direct study as a proxy for clinical judgment. Prior work in Japan has indicated individual variability in nurses’ deterioration prediction behaviors but did not operationalize interval setting as a primary outcome [21].

We designed a simulation-based observational study to examine how nurse characteristics relate to the next vital sign observation interval immediately after assessing abnormal vitals, focusing on low-urgency and moderate-risk scenarios short of RRS activation thresholds. We further consider how interval setting could be integrated as an explicit learning objective in simulation-based education, aligning with contemporary standards for healthcare simulation and the educational mission to improve early recognition and timely escalation [11,12,13,14,15,22,23,24]. In this study, we did not impose a single “correct” observation interval. Rather, we anchored educational debriefing to reference ranges informed by NEWS2 risk bands and our hospital’s escalation policy, refined through local expert consensus, while measuring nurses’ unconstrained interval-setting behavior as the primary outcome.

## 2. Materials and Methods

Study design and setting: We conducted a prospective exploratory observational study between April and June 2023 at a university hospital in Japan, following STROBE recommendations for reporting observational studies [22]. The institutional ethics committee approved the protocol (Approval No. 6748). Participation was voluntary with written informed consent, and anonymity and the right to withdraw without disadvantage were assured. This study was designed to generate effect-size estimates and educational insights; therefore, analyses were limited to prespecified comparisons without post hoc expansions.

Participants: Eligible participants were registered nurses working on inpatient wards. Nurse managers and assistant managers were excluded to focus on bedside decision-making roles. Recruitment occurred via poster advertisements. Twenty-seven nurses consented and completed all study procedures.

Simulation environment and scenarios: We used a full-body adult wireless patient simulator (SimMan Essential, Laerdal Medical, Stavanger, Norway), which allowed visual and tactile assessment of respiratory rate and pulse. We used a full-body adult wireless patient simulator (SimMan Essential, Laerdal Medical), which allowed visual and tactile assessment of respiratory rate and pulse. Three scenarios with static vital signs represented increasing severity short of rapid response activation thresholds: normal (RR 16/min, HR 80/min), low-urgency (RR 20/min, HR 100/min), and moderate-risk (RR 24/min, HR 120/min).

No formal pre-session calibration protocol was implemented due to time and resource constraints; the simulator underwent routine vendor maintenance and daily function checks. Before each session, we cross-checked programmed respiratory and heart rates against device displays, verified respiratory movements and palpable pulses via visual inspection and palpation, and documented any discrepancies. Future studies will adopt standardized calibration checklists and logs.

The moderate-risk scenario reflects moderately abnormal vital signs relative to commonly used NEWS2 risk bands, providing an educationally meaningful step-up in monitoring needs without mandating team activation. Scenarios were presented in fixed order (normal, low-urgency, moderate-risk) to allow a brief practice run before abnormal scenarios; potential learning or anchoring effects are acknowledged in the Limitations. We designed scenarios and procedures in accordance with the INACSL Healthcare Simulation Standards of Best Practice for Simulation Design [24].

Measures and procedures: Participants first measured vital signs in the normal scenario as a practice session. They then measured vital signs in the low-urgency and moderate-risk scenarios in sequence. After each measurement, participants were asked to specify, in hours (half-hour increments permitted), the interval they considered appropriate for the next observation of vital signs. The instruction emphasized that participants should consider overall patient risk and ward context as they normally would when deciding a monitoring plan, short of activating an RRS.

We collected nurse characteristics that, based on prior literature, might influence deterioration prediction behaviors [21]. These included years of clinical experience (continuous and dichotomized at the sample median for descriptive comparisons), completion of advanced life support (ALS) training, prior experience responding to clinical deterioration, and prior experience recognizing deterioration. In this study, clinical deterioration was defined as the development of a sudden, life-threatening condition requiring urgent intervention. Experience responding to deterioration was defined as having provided urgent interventions to prevent life-threatening outcomes, including when responding as additional support. Experience recognizing deterioration was defined as having identified that a patient had developed a sudden life-threatening condition. ALS was defined in line with international practice as team-based cardiopulmonary resuscitation for patients with severe respiratory or circulatory dysfunction under physician oversight, using medical devices and drugs [25,26]. No formal pre-session device calibration protocol was implemented beyond routine setup; we note this as a limitation related to measurement fidelity.

Statistical analysis: We summarized participant characteristics descriptively and used Mann–Whitney U tests for univariate comparisons, reporting Cliff’s delta with 95% confidence intervals as effect size measures. Multiple linear regression models examined adjusted associations with four a priori predictors, reporting unstandardized coefficients, 95% confidence intervals, standard errors, t-statistics, *p*-values, and model R^2^. As sensitivity analyses, we fitted linear mixed-effects models pooling the two abnormal scenarios with participant random intercepts and scenario as fixed effect. We also conducted ordinal logistic regression for the moderate-risk scenario. Model diagnostics included residual and Q–Q plots, variance inflation factors (VIFs) [all <2.0], and HC3 robust standard errors to assess model assumptions and robustness. Given the exploratory nature with prespecified comparisons, *p*-values are presented unadjusted in the main text; Benjamini–Hochberg FDR-adjusted *p*-values are provided in the Supplementary Materials. Statistical significance was set at *p* < 0.05. Analyses used R version 4.3.1.

## 3. Results

Participant characteristics: All twenty-seven nurses completed the simulations and provided complete data. The median clinical experience was three years (interquartile range 2–8 years). Nine participants (33.3%) reported having completed ALS training. Twenty-three participants (85.2%) had experience responding to clinical deterioration, and twenty participants (74.1%) had experience recognizing deterioration (Table 1). The sample was heavily weighted toward early-career nurses, consistent with local workforce demographics.

**Figure 1:**
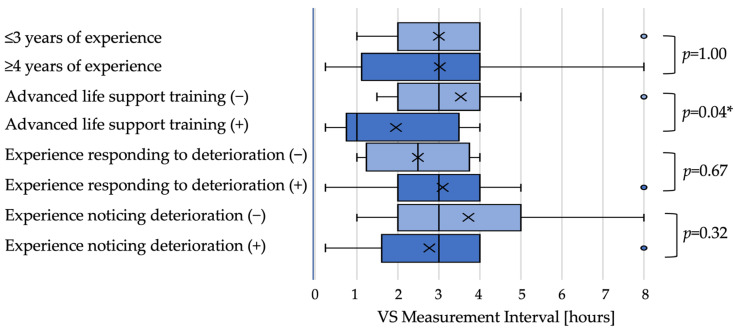
Observation intervals in the low-urgency scenario (box plot). Boxplots of the next vital sign observation interval in the low-urgency scenario by nurse characteristics. Boxes indicate interquartile ranges; horizontal lines indicate medians; whiskers represent the 5th and 95th percentiles. Light blue boxes represent the first category listed for each characteristic (e.g., ‘≤3 years of experience’, ‘Advanced life support training (−)’), while dark blue boxes represent the second category (e.g., ‘≥4 years of experience’, ‘Advanced life support training (+)’). Group medians were 3 h for ≤3 years vs. ≥4 years of experience (*p* = 1.00) and 1 h vs. 3 h for ALS-trained vs. non-trained (*p* = 0.04). “*” indicates statistical significance (*p* < 0.05).

Low-urgency scenario: In the low-urgency scenario, the median next observation interval did not differ by dichotomized experience when examined by medians (≤3 years: 3 h; ≥4 years: 3 h; *p* = 1.00). Nurses with ALS training set shorter intervals than those without training (1 h vs. 3 h; *p* = 0.04). Intervals did not differ significantly by experience responding to deterioration (2.5 h vs. 3 h; *p* = 0.67) or recognizing deterioration (both medians 3 h; *p* = 0.32) (Figure 1).

**Figure 2:**
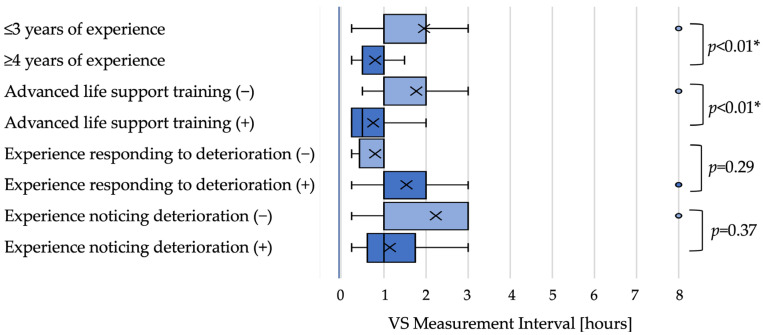
Observation intervals in the moderate-risk scenario (box plot) Boxplots of the next vital sign observation interval in the moderate-risk scenario by nurse characteristics. Shorter intervals were observed among nurses with ≥4 years of experience and those with ALS training in univariate analyses (both *p* < 0.01). Boxes indicate interquartile ranges; horizontal lines indicate medians; whiskers represent the 5th and 95th percentiles. Light blue boxes represent the first category listed for each characteristic (e.g., ‘≤3 years of experience’, ‘Advanced life support training (−)’), while dark blue boxes represent the second category (e.g., ‘≥4 years of experience’, ‘Advanced life support training (+)’). “*” indicates statistical significance (*p* < 0.05).

Moderate-risk scenario: In the moderate-risk scenario, nurses with greater experience tended to set shorter intervals. Although the median interval was 1 h in both groups (≤3 years and ≥4 years), distributions differed with a shift toward shorter intervals among more experienced nurses (*p* < 0.01). Nurses with ALS training also set shorter intervals than those without training (0.5 h vs. 1 h; *p* < 0.01). Intervals did not differ significantly by experience responding to deterioration (both medians 1 h; *p* = 0.29) or recognizing deterioration (both medians 1 h; *p* = 0.37) in univariate tests (Figure 2).

**Figure 3:**
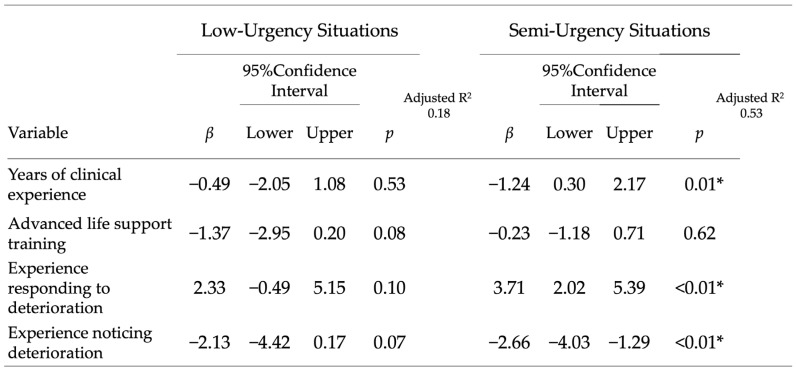
Adjusted associations with observation intervals in the moderate-risk scenario. Multiple regression analysis of factors associated with the next vital sign observation interval in the moderate-risk scenario. Years of clinical experience, prior response to deterioration, and prior recognition of deterioration were independently associated with shorter intervals (all *p* < 0.05), whereas ALS training was not significant after adjustment. The model was prespecified and included four predictors. “*” indicates statistical significance (*p* < 0.05).

Adjusted analyses: In multiple regression models for the low-urgency scenario, no nurse characteristic was independently associated with the next observation interval. In contrast, in the moderate-risk scenario, years of clinical experience, experience responding to deterioration, and experience recognizing deterioration were independently associated with shorter intervals (all *p* < 0.05), whereas ALS training was not significant after adjustment. The adjusted associations are summarized in Figure 3.

Full regression results for the moderate-risk scenario, including unstandardized coefficients, 95% confidence intervals, and model R^2^, are presented in Table 2. The model accounted for 41.9% of variance in observation intervals in the moderate-risk scenario (adjusted R^2^ = 0.318). All variance inflation factors were <2.0, indicating no multicollinearity concerns. Benjamini–Hochberg FDR-adjusted *p*-values for the eight prespecified Mann–Whitney comparisons indicated that experience (*p*_BH = 0.040) and ALS training (*p*_BH = 0.047) remained significant in the moderate-risk scenario after multiple-testing correction. Sensitivity analyses using linear mixed-effects models (with participant random intercepts and scenario as fixed effect) and ordinal logistic regression for the moderate-risk scenario yielded directionally consistent results, supporting the robustness of the main findings. Model diagnostics, variance inflation factors (VIFs), and sensitivity analyses (linear mixed-effects and ordinal logistic models) are provided in the Supplementary Materials.

## 4. Discussion

In a controlled simulation environment, we examined the next vital sign observation interval as a concrete, observable behavior that reflects clinical judgment when abnormal values are detected. Three implications emerge. First, interval setting appears to be shaped by experiential factors: more experienced nurses and those with prior exposure to deterioration set shorter intervals when vital signs are moderately abnormal. This aligns with conceptual models in which experience facilitates earlier cue recognition and more conservative monitoring decisions [19,20]. Second, ALS training was associated with shorter intervals in univariate analyses but not after adjustment in the moderate-risk scenario, suggesting that acute resuscitation training alone may not explain interval setting once clinical experience and exposure to deterioration are considered. Third, the decision itself is measurable and teachable; integrating interval setting as a learning objective in simulation provides a practical lever for strengthening the “assessment-to-action” link in early deterioration recognition.

Our findings resonate with the real-world observation that monitoring frequency can diverge from protocol recommendations as risk rises, likely reflecting the interplay of workload, risk perception, and clinical judgment [12]. Electronic NEWS2 implementations can shorten time to the next observation but do not consistently alter human decision patterns [13], and the integration of scores with structured assessment has been advocated to guide monitoring and escalation [14]. In this context, interval setting can serve as a proximal, observable outcome for educational interventions that aim to calibrate judgment against risk. For instance, in a moderate-risk scenario, it may be reasonable to target intervals of thirty to sixty minutes coupled with explicit contingency plans, whereas in a low-urgency scenario, intervals of one to three hours may be acceptable depending on the broader clinical picture and ward workload. Framing these targets within NEWS2 risk bands and local escalation policies provides a common language for feedback and debriefing [11,15,16,17]. While our simulation isolated clinical judgment from contextual constraints, real-world monitoring intervals are continuously negotiated against workload, competing demands, and resource limitations. This human factor context likely contributes to persistent variability in interval-setting behavior observed after electronic NEWS2 implementations and underscores the need to support clinical judgment within ward work realities.

The pattern observed for clinical experience likely reflects both time-in-role and exposure to shift leadership responsibilities. In Japan, many institutions expect nurses to prepare for shift leader roles around the third year of practice, with curricula emphasizing situational awareness, team coordination, and escalation. These responsibilities may sharpen anticipatory monitoring behaviors, including choosing shorter observation intervals when faced with compounded abnormalities [27,28]. The association between prior exposure to deterioration and shorter intervals may similarly capture the effect of salient clinical events on risk perception. Nurses who have personally recognized deterioration often describe heightened vigilance and a propensity to “double-check” sooner, whereas those who typically respond as additional team members may focus more on procedural tasks once escalation is underway [29]. While our adjusted analyses linked both recognition and response experiences to shorter intervals, the narrative remains that personal proximity to the first signs of deterioration can imprint future monitoring decisions. Our findings suggest that experiential differences—years of practice and exposure to deterioration—represent plausible sources of variability in interval-setting, a key decision point between assessment and action. Future studies should incorporate think-aloud protocols or cognitive task analysis during interval setting to illuminate the cues and heuristics underlying these clinical judgments. Additionally, the moderate within-nurse consistency across scenarios (intraclass correlation = 0.384, 95% CI [0.062, 0.650]) suggests that individual nurses show only moderate agreement between low-urgency and moderate-risk scenarios. This finding indicates that judgment about appropriate intervals is not a fixed trait but rather context-dependent, varying with perceived patient acuity and clinical complexity. This reinforces the importance of understanding how nurses adapt their monitoring decisions to real-world clinical contexts and suggests that educational interventions should emphasize flexible, risk-responsive judgment rather than rigid protocols.

Beyond scores alone, interval setting is an observable bridge between assessment and action. Making “the next observation interval” an explicit learning objective operationalizes clinical judgment by asking learners to state a concrete interval, articulate the rationale integrating early warning scores with patient context, and specify contingencies (for example, what earlier signs would trigger escalation). Faculty can then compare chosen intervals with local policies and commonly accepted ranges for low-urgency versus moderate-risk abnormalities, discuss cognitive biases (such as normalcy bias after a practice scenario), and reinforce timely monitoring and escalation behaviors. These patterns align with Tanner’s model of clinical judgment, in which experience supports cue recognition and timely responding, and with Benner’s novice-to-expert framework, where anticipatory monitoring evolves with practice [19,20]. Interval setting maps directly onto the “responding” phase and provides material for “reflecting” during debriefing.

## 5. Limitations

Several limitations warrant consideration. First, this single-center, exploratory study involved a small, early-career–skewed sample (median experience 3 years), which limits generalizability and statistical power; findings should be interpreted as hypothesis-generating.

Second, scenarios were presented in a fixed order (normal → low-urgency → moderate-risk), introducing potential learning or anchoring effects. Because all participants experienced the same sequence, order effects are inseparable from scenario severity and could not be analyzed independently. A randomized crossover design with adequate washout periods is warranted in future work.

Third, we did not collect unit specialty, educational background, or self-efficacy measures, which limits interpretation and may confound associations. Prior deterioration experiences were captured as self-reported binary variables, not reflecting recency, intensity, or emotional salience.

Fourth, analyses were scenario-specific and did not model within-subject correlation; the moderate intraclass correlation (ICC = 0.384) suggests judgment may vary across contexts. Fifth, although we verified simulator outputs through cross-checking and visual/tactile confirmation, we lacked formal pre-session calibration protocols; future work will implement standardized calibration procedures.

Finally, we report planned *p*-values without post hoc correction in the main text, consistent with the exploratory nature of the study; Benjamini–Hochberg FDR-adjusted *p*-values are provided in Supplement Table S3 for sensitivity analysis, with experience and ALS training remaining significant in the moderate-risk scenario after multiple-testing correction (*p*_BH = 0.040 and 0.047, respectively). Future work should include larger multicenter samples stratified by unit type, randomized scenario order, mixed-effects modeling, and richer statistical reporting.

## 6. Conclusions

In a simulation-based observational study, nurses’ decisions about the next vital sign observation interval after detecting abnormal values were associated with clinical experience and prior exposure to deterioration, particularly in moderate-risk scenarios. ALS training was linked to shorter intervals in unadjusted comparisons but was not independently associated after controlling for experience variables. Defining “the next observation interval” as a learning objective offers an actionable way to strengthen pre-deterioration clinical judgment and align educational feedback with early warning systems and local escalation policies. Given the exploratory design, small sample, and fixed scenario order, these findings should be viewed as hypothesis-generating and used to inform larger confirmatory studies and curricular implementation. These single-center, small-sample findings are hypothesis-generating and intended to inform larger confirmatory studies and curricular design.

## Figures and Tables

**Table 1:** Participant characteristics (*n* = 27).

Variable	*N* = 27
Years of clinical experience Median [25–75 percentile]	3.0 [2.0–8.0]
Advanced life support (ALS) training experience	
Yes	9 (33.3)
No	18 (66.7)
Experience in responding to deterioration	
Yes	23 (85.2)
No	4 (14.8)
Experience in noticing deterioration	
Yes	20 (74.1)
No	7 (25.9)

Summary of nurse demographics and professional experience. Continuous variables are shown as median [25th–75th percentile]; categorical variables as *n* (%). Abbreviations: ALS, advanced life support.

**Table 2:** Multiple linear regression for next observation interval in moderate-risk scenario (*n* = 27).

Predictor	*β*	SE	t	*p*	95% CI
Intercept	2.447	0.489	5.005	<0.001	[1.437, 3.457]
Years of experience	−0.084	0.038	−2.211	0.038	[−0.163, −0.005]
ALS training (yes = 1)	−0.325	0.372	−0.874	0.392	[−1.090, 0.440]
Response experience (yes = 1)	−1.063	0.476	−2.233	0.037	[−2.043, −0.083]
Recognition experience (yes = 1)	−0.797	0.361	−2.207	0.039	[−1.543, −0.051]

Model R^2^ = 0.419, Adjusted R^2^ = 0.318, F(4,22) = 3.963, *p* = 0.016. Abbreviations: SE, standard error; CI, confidence interval.

## Data Availability

Due to privacy and ethical restrictions, de-identified data can be shared upon reasonable request to the corresponding author for research purposes. Analysis code and study materials are also available upon request.

## Supplementary Materials

The following supporting information can be downloaded at: https://www.mdpi.com/article/10.3390/nursrep15120416/s1, Table S1: Variance Inflation Factors (VIF) and Model Fit Statistics; Table S2. Effect Sizes (Cliff’s Delta) for Mann-Whitney U Comparisons; Table S3. Benjamini-Hochberg FDR-Adjusted *p*-Values (8 Prespecified Mann-Whitney Comparisons); Table S4. Linear Mixed-Effects Model Results: Pooled Analysis of Low-Urgency and Moderate-Risk Scenarios (n = 27, 54 observations); Text S1. Post-Hoc Detectability Analysis; Text S2. Intraclass Correlation and Judgment Consistency.

## Public Involvement Statement

There was no public involvement in any aspect of this research.

## Guidelines and Standards Statement

This manuscript was drafted against the (STROBE) for prospective observational research.
