# Supplementary material for: Setting the Next Vital Sign Observation Interval as a Learning Objective in Simulation-Based Nursing Education: A Prospective Exploratory Observational Study
Source: Nurs Rep. 2025 Nov 26;15(12):416. doi: 10.3390/nursrep15120416 (PMC12736265; doi:10.3390/nursrep15120416)
Supplement: Supplementary file 1 [file nursrep-15-00416-s001.zip › nursrep-3955784-supplementary.pdf]

Supplement Table S1. Variance Inflation Factors (VIF) and Model Fit Statistics

Predictor	VIF	Tolerance
Years of experience	1.247	0.802
ALS training	1.156	0.865
Response experience	1.089	0.919
Recognition experience	1.302	0.768

Model $R^2 = 0.419$, Adjusted $R^2 = 0.318$, $F(4,22) = 3.963$, $p = 0.016$.

Note: All VIF < 2.0 indicates no multicollinearity concerns.

Supplement Table S2. Effect Sizes (Cliff's Delta) for Mann-Whitney U Comparisons

Scenario	Comparison	Cliff's d	95% CI	Interpretation
Low-urgency	Experience (≤3 vs. ≥4)	0.000	[-0.333, 0.333]	Negligible
Low-urgency	ALS (No vs. Yes)	-0.370	[-0.667, -0.037]	Small
Low-urgency	Response (No vs. Yes)	0.118	[-0.393, 0.554]	Negligible
Low-urgency	Recognition (No vs. Yes)	0.143	[-0.286, 0.500]	Negligible
Moderate-risk	Experience (≤3 vs. ≥4)	-0.489	[-0.733, -0.178]	Medium
Moderate-risk	ALS (No vs. Yes)	-0.407	[-0.667, -0.074]	Small-Medium
Moderate-risk	Response (No vs. Yes)	-0.147	[-0.554, 0.393]	Negligible
Moderate-risk	Recognition (No vs. Yes)	-0.143	[-0.500, 0.286]	Negligible

Note: Cliff's d ranges from -1 (no overlap) to +1 (complete overlap). Interpretation: $|d| < 0.147$ = negligible, 0.147–0.330 = small, 0.330–0.474 = medium, > 0.474 = large.

Supplement Table S3. Benjamini-Hochberg FDR-Adjusted P-Values (8 Prespecified Mann-Whitney Comparisons)

Test	Unadjusted p	Rank	BH-Adjusted p	Significant (α=0.05)?
interval1_Experience	1.000	8	1.000	No
interval1_ALS	0.040	1	0.320	No
interval1_Response	0.670	7	1.000	No
interval1_Recognition	0.320	5	1.000	No
interval2_Experience	0.005	2	0.040	Yes*
interval2_ALS	0.007	3	0.047	Yes*
interval2_Response	0.290	6	1.000	No

interval2_Recognition	0.370	4	0.925	No

*Significant after FDR correction at α = 0.05

Supplement Table S4. Linear Mixed-Effects Model Results: Pooled Analysis of Low-Urgency and Moderate-Risk Scenarios (n=27, 54 observations)

Predictor	Coefficient	SE	t	*p*	95% CI
Intercept	1.892	0.412	4.593	<0.001	[1.073, 2.711]
Scenario (moderate vs. low)	-0.645	0.287	-2.248	0.029	[-1.218, -0.072]
Years of experience	-0.062	0.031	-2.000	0.051	[-0.123, 0.000]
ALS training	-0.287	0.301	-0.954	0.345	[-0.886, 0.312]
Response experience	-0.756	0.387	-1.955	0.056	[-1.535, 0.023]
Recognition experience	-0.542	0.293	-1.850	0.071	[-1.126, 0.041]

Random Effects:

Participant (intercept) variance: 0.234

Residual variance: 1.156

Model fit: AIC = 156.3, BIC = 179.5

Note: Results are directionally consistent with scenario-specific analyses.

Supplement Text S1. Post-Hoc Detectability Analysis

Given the small sample size (n=27) and exploratory design, we estimated the minimal detectable effect size (MDE) for key contrasts using a two-tailed t-test with α=0.05 and power=0.80:

For the experience comparison (n1=15, n2=12): MDE (Cohen's d) ≈ 0.82 (medium-to-large effect)

For the ALS comparison (n1=18, n2=9): MDE (Cohen's d) ≈ 0.95 (large effect)

The observed effect sizes (Cliff's d) for significant findings in the moderate-risk scenario ranged from -0.407 to -0.489, which approach or exceed these thresholds. This suggests the study was adequately powered to detect the observed associations in the

moderate-risk scenario, but would require larger samples to detect smaller effects. These findings should be interpreted as hypothesis-generating and confirmed in larger studies with adequate power for primary outcomes.

Supplement Text S2. Intraclass Correlation and Judgment Consistency

To assess within-nurse consistency across scenarios, we calculated the intraclass correlation (ICC) between interval1 (low-urgency) and interval2 (moderate-risk) responses.

ICC(2,1) = 0.384, 95% CI [0.062, 0.650], $p = 0.034$
Pearson correlation: $r = 0.412$ ($p = 0.034$)

This moderate ICC suggests that individual nurses showed moderate consistency in their interval-setting decisions across the two scenarios. The moderate-to-low consistency may reflect:

(1) Scenario-dependent judgment: Nurses calibrate intervals to perceived risk level.
(2) Contextual variability: Workload or acuity mix influences decisions.
(3) Measurement variation: Random variation or measurement error.

This finding reinforces the importance of contextual factors and suggests that interval-setting is not a fixed trait but rather a dynamic judgment influenced by perceived patient acuity and clinical context. Educational interventions should emphasize flexible, risk-responsive judgment rather than rigid protocols.
